# LEVERAGING VIDEO CONFERENCING TECHNOLOGY TO FACILITATE SOCIAL ENGAGEMENT IN OLDER ADULTS

**DOI:** 10.1093/geroni/igac059.2188

**Published:** 2022-12-20

**Authors:** George Mois, Elizabeth Lydon, Shraddha Shende, Madina Khamzina, Dillon Myers, Raksha Mudar, Wendy Rogers

**Affiliations:** University of Illinois Urbana-Champaign, Urbana Champaign, Illinois, United States; University of Illinois Urbana-Champaign, Champaign, Illinois, United States; University of Illinois Urbana Champaign, Champaign, Illinois, United States; University of Illinois Urbana Champaign, Champaign, Illinois, United States; OneClick, Philadephia, Pennsylvania, United States; University of Illinois Urbana Champaign, Champaign, Illinois, United States; University of Illinois Urbana Champaign, Champaign, Illinois, United States

## Abstract

Social engagement is critical for maintaining well-being and quality of life in older adults. However, typical age-related changes across bio-psychosocial dimensions as well as age-related conditions such as mild cognitive impairment (MCI), pose many challenges for older adults to remain socially connected. The development of technologies, specifically video conferencing, can be leveraged as a tool to facilitate the delivery of social engagement opportunities, both enhancing access and minimizing social isolation. For successful adoption of these tools, the needs and preferences of older adult users must be considered. We employed an iterative research process for designing and developing a social-engagement intervention using a platform called OneClick.chat. We provide community-dwelling older adults with opportunities to engage in casual conversations and reminisce with others on topics of shared interests (e.g., nature, food, hobbies). Our iterative design process involved an interdisciplinary team of engineers, human factors specialists, gerontologists, and neuropsychologists. We also evaluated the system with eight participants (aged 50-64) who provided their insights pertaining to the content, delivery, perceived ease of use, usefulness, and potential adoption of this video-conferencing platform. Based on the feedback received, we have optimized the intervention in preparation for a randomized controlled trial. Furthermore, we provide key insights related to the implementation of social engagement through video technologies with the aim of facilitating social connectivity. Our learnings can be used to guide future work involving video-technology-based interventions to facilitate social connectivity for older adults with varying cognitive abilities.

